# Ozone and PM_2.5_ Exposure and Acute Pulmonary Health Effects: A Study of Hikers in the Great Smoky Mountains National Park

**DOI:** 10.1289/ehp.8637

**Published:** 2006-02-09

**Authors:** Steven P. Girardot, P. Barry Ryan, Susan M. Smith, Wayne T. Davis, Charles B. Hamilton, Richard A. Obenour, James R. Renfro, Kimberly A. Tromatore, Gregory D. Reed

**Affiliations:** 1 Department of Chemistry and; 2 Rollins School of Public Health, Emory University, Atlanta, Georgia, USA; 3 Department of Instructional Technology, Health, and Educational Studies and; 4 Department of Civil and Environmental Engineering, University of Tennessee, Knoxville, Tennessee, USA; 5 University of Tennessee Graduate School of Medicine, Knoxville, Tennessee, USA; 6 National Park Service, Great Smoky Mountains National Park, Gatlinburg, Tennessee, USA

**Keywords:** air pollution epidemiology, fine particulate matter exposure, Great Smoky Mountains National Park, ozone exposure, pulmonary function, spirometry

## Abstract

To address the lack of research on the pulmonary health effects of ozone and fine particulate matter (≤ 2.5 μm in aerodynamic diameter; PM_2.5_) on individuals who recreate in the Great Smoky Mountains National Park (USA) and to replicate a study performed at Mt. Washington, New Hampshire (USA), we conducted an observational study of adult (18–82 years of age) day hikers of the Charlies Bunion trail during 71 days of fall 2002 and summer 2003. Volunteer hikers performed pre- and posthike pulmonary function tests (spirometry), and we continuously monitored ambient O_3_, PM_2.5_, temperature, and relative humidity at the trailhead. Of the 817 hikers who participated, 354 (43%) met inclusion criteria (nonsmokers and no use of bronchodilators within 48 hr) and gave acceptable and reproducible spirometry. For these 354 hikers, we calculated the posthike percentage change in forced vital capacity (FVC), forced expiratory volume in 1 sec (FEV_1_), FVC/FEV_1_, peak expiratory flow, and mean flow rate between 25 and 75% of the FVC and regressed each separately against pollutant (O_3_ or PM_2.5_) concentration, adjusting for age, sex, hours hiked, smoking status (former vs. never), history of asthma or wheeze symptoms, hike load, reaching the summit, and mean daily temperature. O_3_ and PM_2.5_ concentrations measured during the study were below the current federal standards, and we found no significant associations of acute changes in pulmonary function with either pollutant. These findings are contrasted with those in the Mt. Washington study to examine the hypothesis that pulmonary health effects are associated with exposure to O_3_ and PM_2.5_ in healthy adults engaged in moderate exercise.

Both observational studies and controlled-chamber studies have been used to assess acute effects of air pollution on lung function in adults engaged in exercise or work (Aris et al. 1991; Avol et al. 1984; Brunekreef et al. 1994; Folinsbee et al. 1984, 1988; Gong et al. 1986; Hazucha 1987; Horstman et al. 1990; Kinney et al. 1996; Korrick et al. 1998; McBride et al. 1994; McDonnell et al. 1993, 1995, 1997; Naeher et al. 1999; Pekkanen et al. 2002; Selwyn et al. 1985; Spektor et al. 1988; Torres et al. 1997). Although fewer in number, observational studies offer the advantage of studying the effects of pollution on humans engaged in “real-world” activities in natural settings (Thurston and Ito 2001). However, they also have significant methodologic challenges. These include *a*) identifying an accessible population at risk whose exposures can be defined and adequately characterized, *b*) specifying measurable health outcomes, *c*) collecting an adequate amount of suitable quality-assured data on exposure and health outcomes, *d*) collecting sufficient data on other factors that may influence the exposure–outcome relationship, and *e*) the logistical issues of employing properly trained and motivated field technicians, finding cooperative subjects, and having a large enough sample size to adequately power the statistical analyses (Lippmann 1989).

In 1992 and 1993, Harvard University researchers performed a large observational study of day hikers at Mt. Washington in the White Mountain National forest of New Hampshire (Korrick et al. 1998). The Mt. Washington area is a popular site for outdoor recreation but is plagued with episodically high levels of ozone and fine particulate matter (≤ 2.5 μm in aerodynamic diameter; PM_2.5_) due to transported air pollutants and their precursors from surrounding industrial and urban areas (Korrick et al. 1998). Among the significant findings in the study were a 2.2% decline (*p* = 0.003) in forced vital capacity (FVC) and a 2.6% decline (*p* = 0.02) in forced expiratory volume in 1 sec (FEV_1_) for each 50 ppbv (parts per billion by volume) increment in mean O_3_ and consistent associations of decrements in both FVC (0.4% decline, *p* = 0.001) and peak expiratory flow (PEF; 0.8% decline, *p* = 0.05) across the interquartile range for PM_2.5_ concentration of 9 μg/m^3^ after adjusting for age, sex, smoking status, history of asthma or wheeze, hours hiked, ambient temperature, and other covariates.

The Great Smoky Mountains National Park is also a popular outdoor recreation area where ongoing monitoring has revealed high levels of air pollutants. Located in the southern Appalachian Mountains, the park encompasses 2,100 km^2^ (520,000 acres) on the border of western North Carolina and eastern Tennessee. Approximately 95% of this acreage is forested, and elevations range from 267 to 2,021 m. With an average of > 8 million annual visitors since 1990, the park is one of the nation’s most popular. Unfortunately, it also experiences levels of O_3_ and PM_2.5_ that exceed those in any other national park in the eastern United States and often exceed those in nearby cities (National Park Service Air Resources Division 2002). As of 2004, the entire park was classified by the U.S. Environmental Protection Agency (EPA) as a nonattainment area for the 8-hr National Ambient Air Quality Standard (NAAQS) of 80 ppbv, and a portion of the park was classified as nonattainment for the 24-hour PM_2.5_ NAAQS of 65 μg/m^3^ (National Park Service Air Resources Division 2005). Furthermore, between 1990 and 2003, the Great Smoky Mountains was one of six national parks or federal lands to experience statistically significant increases in O_3_ (U.S. EPA 2004b). As with the Mt. Washington area, the cause of these air quality problems is primarily the regional transport of air pollutants and their precursors from nearby metropolitan areas. For the Smoky Mountains, these areas include North Carolina, Georgia, Ohio, and Tennessee (National Park Service Air Resources Division 2002; Renfro 2002). Transported pollutants may then be sustained at elevated levels at higher elevation (> 1,000 m) sites, due primarily to geography and the lack of sources of nitric oxide to promote O_3_ titration (Aneja and Li 1992; Malone 2003).

As a class I area protected under the federal Clean Air Act (1990), the park has air quality issues that have received much attention from the popular media (Barringer 2004), advocacy groups (National Parks Conservation Association 2004), the U.S. Congress (U.S. General Accounting Office 2001), and multi-organizational research efforts (Southern Appalachian Mountains Initiative 2002; Southern Oxidants Study 2002). Despite this attention, to our knowledge, no formal studies have been conducted in the park to document the possible health impacts of air pollution on people who recreate there.

To address this lack of research and to add to the epidemiologic literature on acute health effects of air pollution, we assessed the effects of O_3_ and PM_2.5_ on the pulmonary function of hikers at a popular recreation site in the park. Specifically, our primary goals were to determine whether the high levels of O_3_ and PM_2.5_ frequently observed in the Great Smoky Mountains National Park were associated with decrements in lung function of adult day hikers and to compare these findings with those reported in the Mt. Washington study.

## Materials and Methods

We conducted an epidemiologic study of day hikers of the Charlies Bunion trail on 71 days over two periods: 10 August 2002 through 16 October 2002 (29 sampling days) and 17 June 2003 through 27 August 2003 (42 sampling days). The Charlies Bunion trail is an approximately 6.7 km portion (one-way) of the Appalachian Trail originating at Newfound Gap, a popular high-elevation (1.54 km) destination in the Great Smoky Mountains National Park.

Between 0900 and 1200 hr, we solicited adult (≥18 years of age) volunteers embarking on day hikes along the Charlies Bunion trail to participate in the study. In accordance with all federal guidelines governing use of human participants, we obtained written informed consent from those volunteers choosing to participate. This informed consent procedure was overseen by institutional review boards at both the University of Tennessee and Emory University. A participating hiker was then assigned a random four-digit code, and we obtained height and weight (with and without any hiking load) data. All researchers involved in data collection and analysis completed the National Institutes of Health *Human Participant Protections Education for Research Teams* online course (National Institutes of Health 2006) and any additional human subject protection education programs required by their respective universities’ institutional review boards. Data collection days were rotated between two teams: one led by the University of Tennessee and one led by Emory University and Western Carolina University.

### Pulmonary function testing (spirometry)

To assess change in pulmonary function, we asked participants to perform spirometry both before their hike and when they returned from their hike. Spirometry technicians received 1–2 days of training by a licensed respiratory therapist in all aspects of performing spirometry. As part of this training, technicians were required to demonstrate proper techniques with mock volunteers and were trained by the respiratory therapist before being allowed to work on the study. Puritan-Bennett Renaissance II Spirometry Systems (Tyco Healthcare, Pleasanton, CA) were used to perform all spirometry.

Prehike pulmonary function tests were typically performed in the mornings (0900–1200 hr), and posthike tests were performed in the afternoons (1400–1900 hr) within 20 min of a hiker’s return to the Newfound Gap trailhead. All tests were performed at 1.54 km above mean sea level inside a retrofitted research van that was equipped with two spirometry stations. Participants were tested in the seated position wearing nose clips and performed a minimum of three and a maximum of eight FVC maneuvers as recommended by the American Thoracic Society (ATS) standards (ATS 1995). Participants were required to have pre-and posthike testing performed by the same technician on the same machine.

On each sampling day, the spirometers were calibrated in the morning before prehike testing and in the afternoon before posthike testing using a fixed-volume, 3-L syringe. Tolerance limits for acceptable calibration were ± 3% (2.91–3.09 L) in accordance with American Association for Respiratory Care Clinical Practice Guidelines (American Association for Respiratory Care 1996).

To determine whether a hiker’s pre- and posthike pulmonary function tests met the ATS acceptability criteria for inclusion in epidemiologic studies, each maneuver within both the pre- and posthike test sessions was evaluated by a pulmonary physician (R.A.O.). The physician, experienced with spirometry and blinded to the study hypothesis, inspected both the flow-volume and volume-time curves to ensure ATS standards were satisfied. Briefly, current (1994) ATS standards for acceptable spirometry include good start of test (an extrapolated volume of ≤ 5% of the FVC or 150 mL, whichever is greater), no hesitation or false start, a rapid start to rise time, no cough, especially during the first second of the maneuver, and no early termination of exhalation (unless there is no volume change for at least 1 sec or the subject cannot or should not continue to exhale further) (ATS 1995). For each hiker who gave at least two acceptable prehike and at least two acceptable posthike maneuvers, we assessed FVC and FEV_1_ reproducibility criteria set forth by the ATS. These criteria require that the largest two FVC values from among acceptable maneuvers be within 0.2 L of each other and the largest two FEV_1_ values from among acceptable maneuvers be within 0.2 L of each other (ATS 1995).

For each hiker who gave acceptable and reproducible pre- and posthike spirometry, we calculated the percentage change in five spirometric values: FVC, FEV_1_, FEV_1_/FVC, PEF, and mean flow rate between 25% and 75% of the FVC (FEF_25–75%_). Percentage change was defined as 100 times the difference of the posthike value minus the prehike value divided by the prehike value. For FVC and FEV_1_, we used the maximum prehike and posthike values from among those maneuvers that were acceptable and reproducible. Prehike and posthike values of FEV_1_/FVC, PEF, and FEF_25–75%_ were taken from the single acceptable and reproducible maneuver with the maximum sum of FEV_1_ and FVC (ATS 1995).

### Trip log diary

Each participant was given a trip log diary to complete during the hike. Along the Charlies Bunion trail there are four National Park Service signs marking various points. These are the Newfound Gap trail-head, Sweat Heifer Creek Trail (2.7 km from Newfound Gap trailhead), Boulevard Trail turnoff (1.6 km from Sweat Heifer Creek Trail), Ice Water Spring Shelter (0.3 km from Boulevard Trail turnoff), and Charlies Bunion (2.1 km from Ice Water Spring Shelter). We provided digital watches, demonstrated proper technique for taking a pulse (radial or carotid), and instructed hikers to record their time of arrival and 15-sec pulse at designated location on ascent (trailhead to highest destination reached) and then on descent (highest destination reached to trailhead) and to note any special circumstances or deviations from the trail. Hikers were not asked to record respiratory symptoms along the hike.

### Respiratory health symptoms and history questionnaire

After completing posthike spirometry, hikers responded to a modified version of the ATS Division of Lung Disease questionnaire (Ferris 1978). The standardized questionnaire obtained information on respiratory illness symptoms (cough, wheeze, phlegm, shortness of breath), history of respiratory illness (chest injury, heart trouble, bronchitis, pneumonia, pleurisy, pulmonary tuberculosis, hay fever, bronchial asthma), use of a bronchodilator within 48 hr, frequency and intensity of weekly aerobic activity, demographics (race, sex, age, marital status, education level, occupation), smoking status (never, current, former), and smoking history (if applicable).

### O_3_ and PM exposure assessment

Real-time ambient O_3_ and PM_2.5_ concentrations, along with temperature and relative humidity, were monitored on-site at the Newfound Gap trail-head on each study day. One-minute average O_3_ concentrations were measured using a ultraviolet-absorption–based O_3_ monitor (model 202; 2B Technologies, Boulder, Colorado). Dynamic calibration of the monitor was performed at the Knox County, Tennessee, Department of Air Quality Management’s Air Quality Laboratory. We performed co-location studies at the Spring Hill Elementary monitoring site in Knoxville, Tennessee. Finally, because most of the Charlies Bunion trail is under forested canopy, we conducted a series of studies to assess a possible canopy effect—the potential reduction of O_3_ concentration due to vegetative uptake and deposition. The details of these studies are presented elsewhere (Malone 2003). Briefly, the portable O_3_ monitor was used to measure concentrations on the trail (under the canopy) and at the trailhead (outside of the canopy). From these studies, a canopy correction factor was developed for the exposure calculations to ensure that the measured O_3_ concentrations accurately reflected a hiker’s true O_3_ exposure.

A β-attenuation filter-based mass monitor (E-BAM; Met One Instruments, Grants Pass, OR) measured 1-hr average PM_2.5_ concentrations. Co-location studies were performed with a continuous PM_2.5_ monitor (tapered element oscillating microbalance) at the Look Rock monitoring station, and flow, temperature, and system calibrations were performed throughout the study.

The O_3_ monitor was small enough to be attached to the E-BAM, and two 12-V DC batteries connected in parallel provided sufficient power for the monitors to run for at least 12 hr. All data were downloaded from the monitors directly onto a laptop computer.

On days where either portable monitor was not operating, we substituted values from two permanent monitoring stations maintained by the National Park Service: Clingmans Dome for O_3_ (a high-elevation site 6.4 km from Newfound Gap, 2.0 km above mean sea level) and Look Rock for PM_2.5_ (located on the eastern border of the park, 0.80 km above mean sea level). In both cases, we corrected the park’s monitors to equivalent values for Newfound Gap based on correlations obtained from co-location studies. The correlation coefficients ranged from 0.6 to 0.9, indicating that correlations between the portable monitors and permanent monitors were adequate. Monitor failure occurred on approximately 15 sampling days for O_3_ and 7 sampling days for PM_2.5_.

Concentrations for O_3_ and PM_2.5_ were reported as 15-min average concentrations for use in exposure calculations. A time-weighted average pollutant (O_3_ or PM_2.5_) concentration for each hiker was calculated by multiplying the average pollutant concentration in each discreet interval along the hike by the fraction of time spent in that interval. Times spent in each of the interval were taken from the trip log diary data. O_3_ canopy corrections were made for portions of the hike under the forested canopy. In general, a 13% decrease in O_3_ concentration was observed within the canopy (Malone 2003).

Fifteen-minute averages of temperature and relative humidity were measured at the trailhead on each study day, and an overall daily average was computed for use in all statistical models.

### Statistical methods

To obtain an estimate of the relationship between O_3_ and PM_2.5_ exposure and change in pulmonary function, we used multiple linear regression, modeled by ordinary least squares estimation, as our primary method of analysis (PROC GLM; SAS Institute Inc., Cary, NC). The dependent variables in these analyses were the percentage change (posthike from prehike) in each of the five spirometric values: FVC, FEV_1_, FEV_1_/FVC, PEF, and FEF_25–75%_. The two pollutant exposure variables, O_3_ and PM_2.5_, were considered the independent variables in the analysis.

To compare results between our study and the Mt. Washington study, we employed a similar modeling strategy. We fit separate regression models for each of the spirometric values as a function of each pollutant exposure. Both univariate and adjusted models were calculated. For the adjusted models, we selected *a priori* covariates based on those adjusted for in the Mt. Washington study. These included both continuous variables (age, hours hiked, and mean temperature) and categorical variables [sex, smoking status (former vs. never), history of asthma or wheeze symptoms, carrying a backpack, and reaching the summit]. In addition to these models, an adjusted piecewise linear regression model was fit for O_3_ using an inflection point of 40 ppbv to determine whether or not different relationships were observed at higher concentrations.

## Results

### Study population

Over the 71 sampling days, 905 hikers initiated participation in the study. Of these hikers, 79 did not return for the posthike testing and an additional nine withdrew (either during pre- or posthike testing). A total of 817 (90.3%) returned for posthike spirometry testing.

Initial eligibility criteria included adult age (≥18 years), nonsmoker (had never smoked or had not smoked for 1 year before testing), no use of bronchodilator or asthma medication within 48 hr of testing, and day hikers who hiked at least to the Sweat Heifer trail marker. Among the 817 hikers who completed the study, 96 (12%) violated at least one of the initial inclusion criteria, and 721 (88%) were retained for further consideration. The most significant reasons for exclusion were smoking (*n* = 43 current smokers) and use of a bronchodilator within 48 hr of the test (*n* = 34).

Pulmonary function tests of these 721 hikers were then evaluated for inclusion in the analysis population as described previously. Of these hikers, 367 (50.9%) were excluded for failure to provide at least two acceptable and reproducible pre- and posthike pulmonary function tests. The most common reason for spirometric test failure was failure to blow out hard enough or long enough (~ 30%). This resulted in a final sample size for the analysis population of 354 hikers.

Selected demographic data for hikers included in the analysis population as well as those excluded are shown in Table 1. Most hikers were white (96%), never smoked (75%), and had no history of asthma or wheeze (82%). Sex was evenly divided, with a slight majority of females (56%). Age ranged from 18 to 82 years, with mean age of 43 years.

We tested for differences between those excluded due to spirometric test failure and those included in the analysis population using chi-square comparisons for categorical variables and two-sided *t*-tests for continuous variables. These results are shown in Table 1. Statistically significant differences (at the 5% level) were seen in sex (more males excluded) and, as a result, in baseline FEV_1_ and FVC. Otherwise, the excluded hikers did not differ substantially from the analysis population.

### Exposure assessment

O_3_ and PM_2.5_ concentrations were lower than anticipated at the onset of the study, and despite a record of frequent violations in past years, there were no exceedances of the current 8-hr NAAQS (80 ppbv) or the 24-hr standard for PM_2.5_ (65 μg/m^3^) during the study period (U.S. EPA 2004a). The average daily O_3_ concentration measured at the Newfound Gap trail-head on the 71 study days was 52.0 ± 13.4 ppbv with a range of 27.6–79.3 ppbv. The average daily PM_2.5_ concentration was 13.9 ± 8.2 μg/m^3^ with a range of 1.6–38.4 μg/m^3^.

Average daily temperature for the study days ranged from 2.6 to 24.1°C with a mean of 19.2 ± 4.4°C, and average daily relative humidity ranged from 48.2 to 93.9% with a mean of 73.6 ± 10.8%.

We computed O_3_ and PM_2.5_ concentrations for hikers included in the analysis data set (*n* = 354) using each hiker's time–weight average concentration including a correction for time spent under the canopy. (Table 1). O_3_ concentrations ranged from 25.0 to 74.2 ppbv with a group mean of 48.1 ± 12.0 ppbv during exercise. PM_2.5_ concentrations ranged from 0.21 to 41.9 μg/m^3^ with a group mean of 15.0 ± 7.4 μg/m^3^ during exercise. For comparison, concentrations were also computed for excluded hikers and are shown in Table 1.

**Figure 1:**
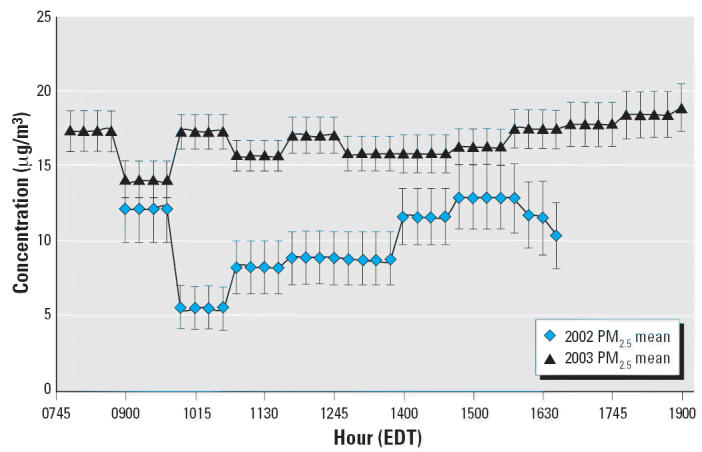
Hourly variation in mean and maximum PM_2.5_ concentration (μg/m^3^) at the Newfound Gap trailhead stratified by sampling period (29 days during August–October 2002 and 42 days during May–August 2003). Times shown are Eastern Daylight Saving Time (EDT).

**Figure 2:**
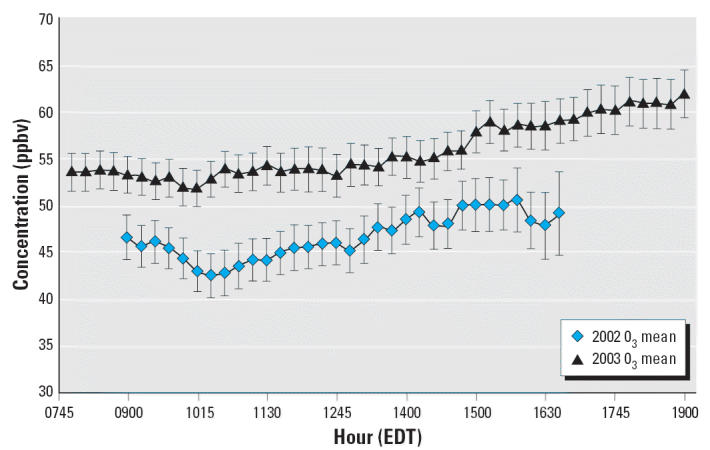
Hourly variation in mean and maximum O_3_ concentration (ppbv) at the Newfound Gap trailhead stratified by sampling period (29 days during August–October 2002 and 42 days during May–August 2003). Times shown are Eastern Daylight Saving Time (EDT).

Figures 1 and 2 show the hourly variation of PM_2.5_ and O_3_, respectively, on study days. In contrast to strong diurnal patterns in urban O_3_, high-elevation sites typically display only small variation in O_3_ concentrations throughout the day (Aneja et al. 2000). These data reflect this high-elevation O_3_ pattern. PM_2.5_ concentrations were also fairly constant throughout the day, with increases in the late afternoon (1500 hr and later). For both pollutants, 2003 levels were slightly higher than those observed in 2002. This was expected because of the seasonal difference between the 2002 and 2003 sampling periods (2002 sampling period was mostly during the fall and 2003 mostly during the summer).

For the 354 included hikers, the mean O_3_ concentrations were significantly (*p* < 0.0001) correlated with mean PM_2.5_ concentrations (Spearman *r* = 0.67). However, both pollutants were weakly but significantly associated with average daily temperature and relative humidity (O_3_: Spearman *r* = 0.16, *p* = 0.0039, and Spearman *r* = –0.59, *p* < 0.0001, respectively; PM_2.5_: Spearman *r* = 0.38, *p* < 0.0001, and Spearman *r* = –0.31, *p* < 0.0001, respectively).

### Exercise profile

From the trip log diaries, we determined each hiker’s highest destination reached, the total hiking distance (using the roundtrip distances from the National Park Service), and the total roundtrip hiking time (defined as time between prehike and posthike spirometry).

Selected exercise characteristics are also summarized in Table 1. Most included hikers (79%) carried a backpack or other load during their hike, with the average load weighing 4.1 ± 2.6 kg. Most (71%) also reached the peak (Charlies Bunion), with the average hiking distance of 12.2 ± 2.4 km and average hiking time of 5.0 ± 1.2 hr. There were no significant differences in the exercise profile compared with excluded hikers.

From the pulse data, we determined each hiker’s maximum self-reported pulse (as number of beats per minute) and the percentage of age-predicted maximum pulse rates achieved, defined as 100 times the maximum self-reported pulse divided by 220 minus the hiker’s age. For hikers included in the study, the mean percent maximum pulse achieved was 68 ± 13% with a range of 35–100%.

We also determined each hiker’s baseline level of physical fitness by asking hikers about their typical exercise intensity and weekly frequency on the ATS-DLD questionnaire. Most (73%) indicated that they exercised at least 2 days per week, and most (72%) indicated that their exercise level was moderate or intense.

### Pulmonary function response to exposure

The crude mean posthike percentage changes in each spirometric variable (FVC, FEV_1_, FEV_1_/FVC, FEF_25–75%_, PEF) were small and, in most cases, positive (Table 2). Only two spirometric variables—PEF and FEV_1_/FVC— had negative overall mean posthike percentage changes: 1.08% and –0.003%, respectively. Crude mean changes for FVC, FEV_1_, and PEF were 0.24%, 0.15%, and 1.27%, respectively.

**Figure 3:**
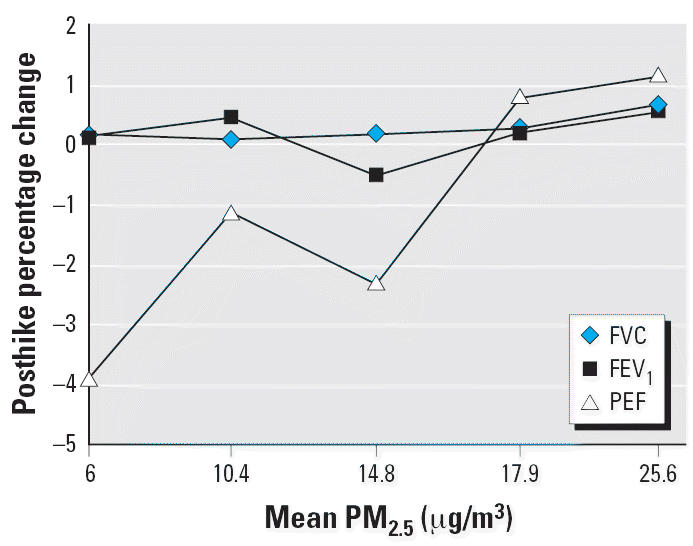
Hikers’ mean posthike percentage change in FVC, FEV_1_, and PEF as a function of quintile of PM_2.5_ concentration.

**Figure 4:**
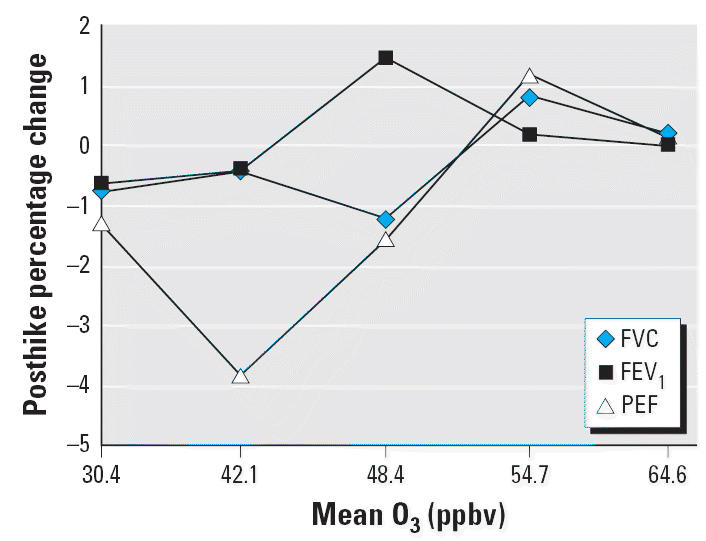
Hikers’ mean posthike percentage change in FVC, FEV_1_, and PEF as a function of quintile of O_3_ concentration.

To explore a possible dose–response relationship between pollutant exposure and pulmonary function, we calculated the quintiles of the observed mean O_3_ and PM_2.5_ distributions and determined the mean posthike percentage change in selected spirometric variables—FVC, FEV_1_, and PEF—within each quintile. These results are summarized in Tables 2 and 3 and displayed graphically in Figures 3 and 4 for PM_2.5_ and O_3_, respectively.

Across the quintiles of O_3_ and PM_2.5_ concentration, the prehike means of each of the pulmonary functions were similar. However, trends in mean posthike percentage changes across quintiles of either pollutant were not statistically significant for any spirometric variable. For FVC and FEV_1_ with O_3_, mean posthike percentage changes were positive with the exception of the first two quintiles (corresponding to O_3_ concentrations of 35.3 and 43.5 ppbv); for FVC and FEV_1_ with PM_2.5_, only quintile 2 (corresponding to a PM_2.5_ concentration of 11.1 μg/m^3^). As Figures 3 and 4 show, the curves for FVC and FEV_1_ are relatively constant, indicating little variation in response as a function of pollutant level.

The PEF response curves show a steady increase from –4.43% to 2.50% across quintiles of PM_2.5_ concentration (Figure 3) and a steady increase from –1.51% to 1.99% across quintiles of O_3_ concentration (Figure 4).

### Multiple linear regression models

Results from multiple linear regression analyses of the percentage change between the pre- and posthike pulmonary function variables (FVC, FEV_1_, FEV_1_/FVC, PEF, and FEF_25–75%_) and the time-weighted average concentration of O_3_ and PM_2.5_ during the hike period are presented in Table 3. Parameter estimates for the exposures, along with their respective *p*-values, are shown for both univariate and adjusted models. In the final adjusted models, we controlled for age, hours hiked, sex, smoking status (never or former), history of asthma or wheeze symptoms, carrying a backpack or other load, reaching the summit, and mean daily temperature. The adjusted models are based on a sample size of *n* = 339 because of missing temperature data for 15 hikers.

In most cases, regression slopes (in units of percent change/concentration) were small and not statistically significant. For example, the coefficient for the percent change in FEV_1_ as a function of PM_2.5_, adjusted for covariates, was 0.003%/μg/m^3^ with a *p*-value of 0.937, indicating that there was no association between PM_2.5_ concentration and change in FEV_1_ over the hike period. Similar interpretations of the coefficients of the other outcome variables and pollutant exposures may be made. Finally, *F*-tests for significant overall regression (data not shown) indicated that the adjusted models did not explain a significant amount of the variation in posthike pulmonary function change. The results from the piecewise model for O_3_ with an inflection point of 40 ppbv did not produce different results. In all cases, except for PEF in the adjusted PM_2.5_ models, the regression slopes were not statistically different from zero.

These conclusions were consistent across several subgroups. There was no change in statistical significance of the regression coefficients for those hikers with a self-reported history of asthma or wheeze (*n* = 62). To improve power, we defined two dichotomous categorical variables based on the ATS-DLD questionnaire responses: a respiratory symptom index based on a hikers’ reporting of any positive symptom of respiratory illness (e.g., cough, cough with phlegm, shortness of breath; *n* = 176) and a respiratory health history index based on whether a hiker reported any positive history of respiratory or cardiovascular illness (e.g., heart trouble, bronchitis, pneumonia, asthma; *n* = 173) (Galizia and Kinney 1999). In both subgroups, mean lung function changes did not differ over the exposure levels, and both univariate and adjusted models resulted in no statistically significant associations. Finally, we restricted analyses to those > 50 years of age (*n* = 103), and our results were the same. We did not perform subanalyses on those with extreme lung function decrements (posthike percentage decrements of ≥ 5% in FVC or FEV_1_) because of lack of sufficient sample (*n* = 40).

To evaluate whether meteorologic variables may have confounded the relationship between exposure and outcome, we computed regression models both with and without average daily temperature and relative humidity. In both cases, results did not change. We included temperature in our final models, however, to compare findings with the Mt. Washington study. We also computed multi-pollutant models, adjusting simultaneously for O_3_ and PM_2.5_. As expected, because of the high correlations between the two pollutants, it was not possible to separate the effects in these models.

### Comparison with the Mt. Washington study

Table 4 compares selected experimental variables between the Mt. Washington and Charlies Bunion (present) studies. The Mt. Washington study was performed on 74 days over 2 years. A total of 766 hikers initiated, with 530 (69%) meeting eligibility criteria. The Charlies Bunion study was performed on 71 days over 2 years. More hikers (*n* = 905) initiated the present study, but the inclusion rate was much smaller (39% compared with 69%). The primary reason for this difference in inclusion was spirometric test failure: fewer subjects in the Charlies Bunion study met ATS requirements for acceptability and reproducibility.

The demographics for both studies were similar. In both, most (96–97%) participants were white, never smokers (71–76%), and had no history of asthma or wheeze (82–92%). The average age was higher in the Charlies Bunion study: 46 compared with 35 in the Mt. Washington study. Finally, males composed a smaller percentage of included subjects (44% in the present study vs. 71% in the Mt. Washington study).

The exercise profile of included hikers in both studies was a significant point of difference. Although there were some similarities, including average maximum pulse rate (122 in the Mt. Washington study vs. 121 in the present study), percentage of age-predicted pulse (66% vs. 68% in the present study), and most reaching the summit and carrying a load, there was a significant difference in exercise (hiking) time. Mt. Washington hikers spent an average of 8 hr hiking, whereas Charlies Bunion hikers spent an average of 5 hr hiking. These differences are reflected in differing exposure levels. Despite similar air pollutant levels in both locations (Mt. Washington vs. Charlies Bunion, respectively: mean O_3_, 40 vs. 47 ppbv; mean PM_2.5_, 15 vs. 15 μg/m^3^), the fact that the Mt. Washington study participants spent more time exercising translated into a higher exposure to pollutants.

Pulmonary function testing between the two studies was similar. In both cases, spirometry was performed in the seated position with nose clips. Posthike testing time was slightly later for the Mt. Washington study because of the longer hike time. One important difference, however, was the coaching. In the Mt. Washington study, only one spirometry technician certified by the National Institute for Occupational Safety and Health (NIOSH) conducted all tests. In the present study, however, 13 technicians were employed. These technicians were predominantly graduate students who had received 1–2 days of training from a certified respiratory therapist. Because spirometry is a highly effort-dependent test, the additional number of technicians may have introduced more variability in the measurements. Finally, baseline values of FEV_1_ and FVC were slightly higher in the Mt. Washington study as a direct result of the larger percentage of males in their analysis population.

Table 5 directly compares selected findings for percentage change in pulmonary function as a function of ambient O_3_ and PM_2.5_ from the two studies. In the Mt. Washington study, adjusted linear models demonstrated statistically significant declines in FEV_1_ (–0.051%/ppbv) and FVC (–0.043%/ppbv) with O_3_ and statistically significant declines in FEV_1_ (–0.041%/μg/m^3^), FVC (–0.043%/μg/m^3^), and PEF (–0.087%/μg/m^3^) with PM_2.5_. In the Charlies Bunion study, linear models adjusting for the same variables did not demonstrate significant associations between posthike change in FEV_1_ and FVC and either pollutant. However, in both studies, there were no significant associations with PEF, FEV_1_/FVC, or FEF_25–75%_ and O_3_.

## Discussion

This study evaluated the hypothesis that exposure to ambient O_3_ and PM_2.5_ leads to acute respiratory effects, as measured by transient changes in pulmonary function, in healthy adults engaged in moderate exercise. Furthermore, we have added to the epidemiologic literature on acute health effects of air pollution by replicating another observational study of healthy adult hikers. To our knowledge, this was one of the first replications of a large-scale observational study of exercising adults. Although there were differences in findings between the two studies, consistent conclusions were reached.

We demonstrated that no statistically significant responses in pulmonary function occur when an average of 5.0 hr of outdoor exercise occurs at the levels of O_3_ and PM_2.5_ that we observed, some of which were substantially below the current NAAQS—80 ppbv for O_3_ (8-hr) and 65 mg/m^3^ for PM_2.5_ (24-hr). Specifically, posthike percentage changes in FVC, FEV_1_, FEV_1_/FVC, FEF_25–75%_, and PEF were not associated with either O_3_ or PM_2.5_ exposure.

In studies where repeated pulmonary function tests are performed within the same day, it is important to assess confounding effects due to diurnal variation in lung function. It has been documented that expiratory flow and volume variables have minimum values early in the morning (0400–0600 hr) and peak around noon (Dockery and Brunekreef 1996). In our study, however, spirometric measurements were made at the same times (prehike, 0900–1200 hr; posthike, 1400–1900 hr) on all study days, regardless of pollution levels. This ensured that this confounding did not occur, but we assessed it quantitatively by computing regression models that were restricted to hikers whose prehike spirometric measurements were taken before 1100 hr and posthike measurement taken after 1500 hr (*n* = 135). Our results did not change.

A potential source of bias in our study was with the spirometry. It has been demonstrated that exclusion of subjects with unacceptable and nonreproducible measurements in studies of pulmonary function and health outcomes may lead to removing subjects with a more accelerated loss of lung function (Eisen et al. 1984). In this study, more than half of the participants were excluded because of spirometric test failure on either the pre- or posthike testing (or both). To assess this potential bias, we performed additional analyses of spirometric test failure using the full study population (*n* = 721). Full descriptions and results of these studies are presented elsewhere (Girardot 2005), but the relevant findings are briefly discussed here. Of the full study population, 700 (97%) hikers provided three complete maneuvers during both the prehike and posthike sessions and were included in these analyses. Spirometric test failure, as defined by the 1994 ATS standards and including both acceptability and reproducibility criteria for the top three maneuvers, was exhibited by 439 (62.7%) participants during prehike sessions and by 424 (60.6%) participants during posthike sessions. For both sessions, reproducibility criteria (both FVC and FEV_1_) for the top two maneuvers were achieved by > 80% of participants (prehike, 84.9%; posthike, 82.3%). Fewer than half of the hikers could perform three acceptable maneuvers during a test session (prehike, 40.3%; posthike, 45.0%), and slightly more could perform at least two acceptable maneuvers during a test session (prehike, 59.7%; posthike, 55.0%). We also sought to examine the association between spirometric test failure and a number of hiker characteristics, including age, sex, body mass index, respiratory health status, and respiratory health history using both stratified analyses and logistic regression modeling, where spirometric test failure was treated as the outcome (coded dichotomously as yes or no). We found no statistically significant associations at the 5% level. Finally, we examined models that included a technician variable as a predictor of test failure. There was no association between technician and spirometric test failure.

These findings imply that the most likely cause of test failure was poor coaching techniques. It has been well argued that achieving quality spirometry depends largely on the “skill and perseverance of the technician” (Enright et al. 2004). In our study, we were faced with the challenge of collecting data from unpaid volunteers in a nonclinical setting (on top of a mountain in a research van) who were generally unfamiliar with the technique and in a hurry to start their hike. Furthermore, we employed graduate students, senior undergraduates, and research assistants. Although they were all trained and approved by a certified respiratory therapist from the University of Tennessee, we realize that coaching volunteer participants—who were frequently uncooperative and/or hesitant—to achieve three acceptable and reproducible maneuvers was extremely difficult. As a result, our recommendations for any field study using spirometry is to employ only NIOSH-certified technicians and to minimize the number of technicians to help reduce the variability that could have been introduced by using different technicians on different days (NIOSH 2004).

Despite the loss of sample size because of poor spirometry, we must point out that the excluded population did not differ substantially from the included population (Table 1). For example, we did not have more hikers with asthma or wheeze excluded because of poor spirometry. In addition, our resulting sample size of *n* = 354 is higher than other studies examining similar hypotheses and is comparable with the Mt. Washington study population of *n* = 530. Finally, before being included in the analyses, each individual maneuver was carefully reviewed by an experienced pulmonary physician (R.A.O.) who was blinded to the study hypothesis. As a result, we feel that the conclusions reached would not differ had more participants been included in the analyses.

There were several additional limitations to our study. First, we could not assess minute ventilation of the hikers to determine a true pollution dose for each hiker. Maximum pulse was used as a proxy for exercise intensity (and hence dose), but this is not an adequate surrogate, because more fit subjects have lower minute ventilation and therefore receive a lower dose of pollutant. In addition, the study did not include children, and there was almost no participation from minority groups such as African Americans or Hispanics. Finally, by choosing to replicate the Mt. Washington study, we were constrained to follow similar protocols and procedures to allow the comparative analysis to be more meaningful. For example, one type of information not considered during this study or in the Mt. Washington study was an assessment of clinical symptoms of respiratory disease during the hike. The ATS, in defining what constitutes an adverse health effect, has stated that reduction in FEV_1_ or FVC must be associated with clinical symptoms (e.g., cough or wheeze) (ATS 2000). Another variable both studies failed to measure was prehiking levels of pollutants. It could be argued that elevated levels of pollutants before the start of a hike might affect the health outcome, especially if these levels were higher than those experienced during the hike. However, we feel that because all of our subjects began their hikes in the morning, when pollution levels are typically at their lowest (even in urban areas), prehike pollution exposure was likely to be minimal. Further, in our study, most hikers arrived in automobiles, which offered some slight protection from air pollution. As a result, we do not feel that this was an issue in either study.

Air quality conditions during the study differed from what was initially predicted based on historical data. During the two study periods, the park had some of the best air quality in many years, due primarily to heavy rainfall. Rainfall “washes out” air pollutants, resulting in good air quality. As a result, the focus of the study shifted from modeling health effects at levels higher than the federal standards to modeling health effects at levels below the current federal standards. The findings from this study directly address the question of whether current federal standards are protective for human health in a healthy, exercising population.

Both this study and the Mt. Washington study examined the respiratory effects of relatively low concentrations of O_3_ and PM_2.5_. One key difference between the two studies was the exposure duration. Mt. Washington hikers averaged 8.0 hr of exercise, whereas hikers in this study averaged 5.0 hr. However, these exercise periods were longer than in many previous field studies, which average exercise times of less than 2 hr. Another key difference was the mean age of the study populations. In the present study, the average age of the hikers was 46 years, compared with 35 in the Mt. Washington study. This is an important point of comparison, because older individuals may be less responsive to O_3_ and PM than younger individuals. Although the Mt. Washington study found significant decrements in FVC and FEV_1_ with both pollutants, the magnitude of the mean changes was small, and as the authors point out, “unlikely to result in clinical symptoms in most individuals” (Korrick et al. 1998). Furthermore, both studies failed to show significant associations in other spirometric variables—PEF, FEV_1_/FVC, or FEF_25–75%_—and O_3_ and between FEV_1_/FVC or FEF_25–75%_ and PM_2.5_. These findings are consistent with previous studies of lung function effects in nonasthmatic subjects. Relatively few observational studies have been conducted on healthy adults engaged in moderate exercise under typical outdoor conditions. For example, results of PM_2.5_ peak flow analyses in several studies reported no consistent evidence for adverse health effects (Vedal 1998).

This study is one of the first designed and conducted, in part, to compare findings from two observational studies of acute respiratory illness and low levels of air pollution in adults engaged in outdoor exercise. Because large-scale observational studies, which are typically expensive and time-consuming to run, are relatively rare, the results obtained from this type of comparative study are important in the epidemiologic literature because they provide evidence (or lack of evidence) of associations between environmental exposure and health effects for individuals in natural settings. Our findings suggest that low levels of pollutant exposure over several hours may not result in significant declines in lung function in healthy adults engaged in exercise or work. However, there is considerable variation in individual response to pollutant exposure, and findings from epidemiologic studies—which rely on testing group means and other indicators—may not be entirely indicative of a lack of individual risk for adverse health effects due to air pollution. Finally, it may be difficult to separate the effects of the exercise or activity itself from the air pollution effects.

## Figures and Tables

**Table 1:** Selected demographic, exercise, and exposure characteristics for included and excluded hikers of the Charlies Bunion Trail in the Great Smoky Mountains National Park, 2002–2003.

Characteristic	Included hikers (*n* = 354)	Excluded hikers (*n* = 367)	*p*-Value*a*
Hike year			
2002	85 (24)	150 (41)	—
2003	269 (76)	217 (59)	—
Demographics			
Race			
White	339 (96)	351 (96)	0.9355
Nonwhite	15 (4)	16 (4)	
Sex (male)	154 (44)	222 (60)	< 0.0001
Age (years)	43.2 ± 12.6 (18–82)	43.3 ± 13.8 (18–82)	0.1108
Smoking status			
Former	90 (25)	103 (28)	0.4232
Never	264 (75)	264 (72)	
Baseline FEV_1_ (L)	3.3 ± 0.77 (1.8–6.5)	3.5 ± 0.82 (1.1–8.5)	0.0079
Baseline FVC (L)	4.3 ± 0.93 (2.0–7. 4)	4.6 ± 0.98 (1.9–9.5)	0.0001
Asthma or wheeze history	62 (18)	52 (14)	0.2184
Exposures			
Mean O_3_ (ppbv) ^b,c^	48.1 ± 12.0 (25.0–74.2)	45.8 ± 12.0 (23.7–74.0)	0.0106
Mean PM_2.5_ (μg/m^3^)^c^	15.0 ± 7.4 (0.21–41.9)	13.3 ± 7.7 (0–41.9)	0.0026
Mean temperature (°C)^d^	20.3 ± 4.2 (2.6–24.1)	19.6 ± 4.0 (2.6–24.1)	0.0250
Mean relative humidity (%)^e^	71.3 ± 10.5 (48.2–93.9)	72.1 ± 11.0 (48.2–93.9)	0.3582
Exercise profile			
Reached summit	251 (71)	270 (74)	0.4242
Carried load	280 (79)	301 (82)	0.321
Mean hike time (hr)^f^	5.0 ± 1.2 (1.8–9.0)	5.0 ± 1.2 (1.8–9.0)	0.4600
Mean hike distance (km)	12.2 ± 2.4 (5.5–25.7)	12.2 ± 2.2 (5.5–25.7)	0.8471

Values shown are mean ± SD (range) or number (%).

a *p*-Values shown compare included hikers with excluded hikers and were computed by chi-square tests for categorical variables and two-sided *t*-tests of means for continuous variables.

b O_3_ concentrations have been corrected for canopy effects.

c Values are based on hiker's time–weight average concentration including a correction for time spent under the canopy.

d Values are based on the average daily temperature on each hiker’s test date.

e Values are based on the average daily relative humidity on each hiker’s test date.

f Defined as time between prehike and posthike pulmonary function tests.

**Table 2:** Mean ± SE of spirometric values in each quintile of PM_2.5_ and O_3_.

Quintile	1 (*n* = 70)*a*	2 (*n* = 71)	3 (*n* = 71)	4 (*n* = 71)	5 (*n* = 71)	Overall (*n* = 354)
PM_2.5_ (μg/m^3^)	6.0	10.4	14.8	17.9	25.6	15.0
Time (hr)*b*	5.0	5.1	5.0	4.9	5.1	5.0
FVC (L)						
Prehike	4.32 ± 0.13	4.30 ± 0.11	4.34 ± 0.12	4.23 ± 0.11	4.15 ± 0.11	4.27 ± 0.05
Posthike	4.33 ± 0.12	4.30 ± 0.11	4.33 ± 0.12	4.23 ± 0.11	4.18 ± 0.12	4.27 ± 0.05
%Δ*c*	+0.12	+0.07	+0.16	+0.23	+0.65	+0.24
FEV_1_ (L)						
Prehike	3.39 ± 0.10	3.42 ± 0.09	3.42 ± 0.10	3.36 ± 0.10	3.31 ± 0.09	3.38 ± 0.04
Posthike	3.40 ± 0.10	3.43 ± 0.09	3.40 ± 0.09	3.36 ± 0.10	3.33 ± 0.10	3.38 ± 0.04
%Δ*c*	+0.13	+0.44	–0.52	+0.18	+0.51	+0.15
FEV_1_/FVC (%)						
Prehike	78.66 ± 0.86	79.36 ± 0.71	79.20 ± 0.81	79.18 ± 0.81	79.73 ± 0.66	79.2 ± 0.34
Posthike	78.63 ± 0.81	79.55 ± 0.69	78.83 ± 0.80	79.26 ± 0.79	79.55 ± 0.64	79.2 ± 0.33
%Δ*c*	+0.07	+0.30	–0.40	+0.17	–0.16	0.003
FEF_25–75%_ (L/sec)						
Prehike	3.27 ± 0.14	3.39 ± 0.14	3.19 ± 0.13	3.34 ± 0.15	3.22 ± 0.14	3.28 ± 0.06
Posthike	3.26 ± 0.14	3.38 ± 0.14	3.21 ± 0.13	3.30 ± 0.15	3.24 ± 0.14	3.28 ± 0.06
%Δ*c*	+1.40	+1.07	+1.05	+2.19	+0.64	+1.27
PEF (L/sec)						
Prehike	7.91 ± 0.22	8.37 ± 0.23	8.12 ± 0.25	7.75 ± 0.25	7.72 ± 0.22	7.97 ± 0.11
Posthike	7.58 ± 0.22	8.26 ± 0.25	7.89 ± 0.25	7.73 ± 0.26	7.77 ± 0.23	7.97 ± 0.11
%Δ*c*	–3.88	–1.14	–2.33	+0.76	+1.12	–1.08
O_3_ (ppbv)	30.4	42.1	48.4	54.7	64.6	48.1
Time (hr)*b*	5.1	4.9	5.0	5.1	5.1	5.0
FVC (L)						
Prehike	4.42 ± 0.13	4.28 ± 0.10	4.24 ± 0.12	4.23 ± 0.10	4.17 ± 0.12	4.27 ± 0.05
Posthike	4.38 ± 0.10	4.26 ± 0.10	4.29 ± 0.12	4.27 ± 0.11	4.18 ± 0.11	4.27 ± 0.05
%Δ*c*	–0.72	–0.40	–1.22	+0.86	+0.24	+0.24
FEV_1_ (L)						
Prehike	3.48 ± 0.11	3.37 ± 0.08	3.34 ± 0.10	3.38 ± 0.08	3.34 ± 0.10	3.38 ± 0.04
Posthike	3.46 ± 0.11	3.35 ± 0.08	3.38 ± 0.10	3.39 ± 0.09	3.33 ± 0.10	3.38 ± 0.04
%Δ*c*	–0.61	–0.40	+1.48	+0.21	+0.05	+0.15
FEV_1_/FVC (%)
Prehike	78.82 ± 0.86	78.70 ± 0.57	78.71 ± 0.96	80.01 ± 0.65	79.86 ± 0.76	79.2 ± 0.34
Posthike	78.89 ± 0.82	78.86 ± 0.57	78.88 ± 0.93	79.51 ± 0.70	79.69 ± 0.68	79.2 ± 0.33
%Δ*c*	+0.19	+0.25	+0.30	–0.64	–0.11	–0.003
FEF_25–75%_ (L/sec)						
Prehike	3.33 ± 0.15	3.20 ± 0.11	3.33 ± 0.15	3.33 ± 0.13	3.23 ± 0.14	3.28 ± 0.06
Posthike	3.35 ± 0.15	3.19 ± 0.11	3.26 ± 0.15	3.32 ± 0.13	3.27 ± 0.15	3.28 ± 0.06
%Δ*c*	+0.34	+1.02	+3.84	–0.06	+1.19	+1.27
PEF (L/sec)						
Prehike	8.32 ± 0.24	7.74 ± 0.21	8.19 ± 0.27	7.87 ± 0.23	7.76 ± 0.24	7.97 ± 0.11
Posthike	8.17 ± 0.14	7.45 ± 0.23	8.03 ± 0.28	7.90 ± 0.24	7.69 ± 0.23	7.85 ± 0.11
%Δ*c*	–1.34	–3.86	–1.56	+1.18	+0.16	–1.08

a Sample size within each quintile of PM_2.5_ or O_3_ concentration.

b Time of exercise defined as difference between prehike and posthike spirometry.

c Percent change defined as 100 times the difference of the posthike value minus the prehike value divided by the prehike value.

**Table 3:** Univariate and adjusted multiple linear regression models of the posthike percentage change in pulmonary function as a function of ambient O_3_ and PM_2.5_.

	FVC	FEV_1_	PEF	FVC/FEV_1_	FEF_25–75%_
PM_2.5_ (univariate)^a^	0.023 ± 0.035 (*p* = 0.51)	0.015 ± 0.029 (*p* = 0.607)	0.185 ± 0.091 (*p* = 0.043)	0.003 ± 0.023 (*p* = 0.905)	0.052 ± 0.093 (*p* = 0.578)
PM_2.5_ (adjusted)^a^^,^^b^	0.007 ± 0.040 (*p* = 0.966)	0.003 ± 0.033 (*p* = 0.937)	0.258 ± 0.103 v	–0.011 ± 0.027 (*p* = 0.676)	–0.041 ± 0.109 (*p* = 0.707)
O_3_ (univariate)^c^	0.015 ± 0.021 (*p* = 0.484)	0.027 ± 0.018 (*p* = 0.145)	0.089 ± 0.057 (*p* = 0.118)	–0.017 ± 0.026 (*p* = 0.525)	–0.051 ± 0.107 (*p* = 0.634)
O_3_ (adjusted)^b^^,^^c^	0.007 ± 0.024 (*p* = 0.763)	0.024 ± 0.020 (*p* = 0.234)	0.118 ± 0.062 (*p* = 0.059)	–0.028 ± 0.016 (*p* = 0.074)	–0.041 ± 0.064 (*p* = 0.523)
O_3_ (piecewise)^b^^,^^c^^,^^d^	–0.019 ± 0.037 (*p* = 0.613)	–0.003 ± 0.032 (*p* = 0.911)	0.127 ± 0.098 (*p* = 0.195)	–0.025 ± 0.025 (*p* = 0.314)	–0.045 ± 0.101 (*p* = 0.659)

a Values shown are β-coefficients for PM_2.5_ exposure in %/μg/m^3^ ± SEs. *p*-Values displayed are for the coefficients.

b Adjusted for age, hours hiked, sex, smoking status (never or former), history of asthma, or wheeze symptoms, carrying a backpack or load, reaching the summit, and mean daily temperature. Because of missing temperature data, *n* = 339 participants were included in these adjusted models.

c Values shown are β-coefficients for O_3_ in %/ppbv ± SEs. *p*-Values displayed are for the coefficients.

d Regression coefficients of piecewise model above inflection point of 40 ppbv O_3_.

**Table 4:** Comparison of selected variables for analysis populations in the Mt. Washington and Charlies Bunion hiker studies.

Characteristic	Mt. Washington	Charlies Bunion
No. initiating study	766	905
No. included for analysis	530	354
Inclusion rate (%)	69	39
No. of study days	74	71
Demographics		
Race [white (nonwhite)]	519 (97)	339 (96)
Sex (male)	375 (71)	154 (44)
Age (years)	35 ± 10 (18–64)	43 ± 9 (19–82)
Tobacco use (never vs. former)	405 (76)	264 (71)
Asthma or wheeze	40 (8)	62 (18)
Exercise profile		
Elevation at trailhead (m above sea level)	620	1,538
Hiking time (hr)	8.0 ± 1.5 (2.0–12.0)	5.0 ± 1.2 (1.8–9.0)
Reached summit	396 (75)	251 (71)
Carried load	498 (94)	280 (79)
Maximum pulse rate (beats/min)	122 ± 26	121 ± 23
Percentage of age-predicted pulse (%)*a*	66 ± 14	68 ± 13
Pulmonary function testing		
Test condition of subjects	Seated with nose clips	Seated with nose clips
Prehike testing time	0800–1030 hr	0800–1200 hr
Posthike testing time	1530–1930 hr	1530–1830 hr
Baseline FEV_1_ (L)	4.08 ± 0.81 (1.82–6.56)	3.38 ± 0.80 (1.83–6.48)
Baseline FVC (L)	5.13 ± 1.02 (2.89–7.93)	4.26 ± 0.97 (1.96–7.45)
Exposures		
O_3_ (ppbv)	40 ± 12 (21–74)	48 ± 12 (25–74)
PM_2.5_ (μg/m^3^)	15 ± 13 (0.7–60)	15 ± 7 (0.2–42)
Trailhead temperature (°C)	17 ± 3 (8–25)	20 ± 4 (3–24)

Values shown are number (%) or mean ± SD (range).

a Defined as maximum self-reported pulse divided by age-predicted theoretical pulse (220-age) times 100%.

**Table 5:** Comparison of multiple linear regression models of the posthike percentage change in pulmonary function as a function of ambient O_3_ and PM_2.5_ in the Mt. Washington (*n* = 530) and Charlies Bunion (*n* = 339) hiker studies.

	O_3_ regression models				PM_2.5_ regression models			
	Univariate		Adjusted*a*		Univariate		Adjusted*a*	
	MW	CB	MW	CB	MW	CB	MW	CB
FEV_1_	–0.045 (*p* = 0.01)	0.015 (*p* = 0.48)	–0.051 (*p* = 0.02)	–0.001 (*p* = 0.29)	–0.035 (*p* = 0.02)	0.023 (*p* = 0.51)	–0.041 (*p* = 0.03)	–0.002 (*p* = 0.96)
FVC	–0.04 (*p* = 0.001)	0.027 (*p* = 0.15)	–0.043 (*p* = 0.003)	0.011 (*p* = 0.08)	–0.038 (*p* = 0.0004)	0.015 (*p* = 0.61)	–0.043 (*p* = 0.0006)	–0.005 (*p* = 0.88)
PEF	–0.033 (*p* = 0.48)	0.089 (*p* = 0.12)	–0.018 (*p* = 0.76)	0.224 (*p* = 0.13)	–0.084 (*p* = 0.02)	0.185 (*p* = 0.043)	–0.087 (*p* = 0.05)	0.282 (*p* = 0.01)
FEV_1_/FVC	–0.005 (*p* = 0.72)	–0.016 (*p* = 0.27)	–0.009 (*p* = 0.61)	–0.023 (*p* = 0.56)	NR	—	NR	—
FEF_25–75%_	–0.005 (*p* = 0.93)	0.006 (*p* = 0.92)	–0.027 (*p* = 0.70)	–0.026 (*p* = 0.48)	NR	—	NR	—

Abbreviations: CB, Charlies Bunion (present) study; MW, Mt. Washington study; NR, not reported.

a Adjusted for age, hours hiked, sex, smoking status (never or former), history of asthma or wheeze symptoms, carrying a backpack or load, reaching the summit, and mean daily temperature.
